# Silent Avascular Necrosis of the Femoral Head in Severe Hemophilia A: A Case Report

**DOI:** 10.7759/cureus.92588

**Published:** 2025-09-17

**Authors:** Anupam Dutta, Mitraa Shyam, Pronami Borah, Subhradeep Biswas

**Affiliations:** 1 Department of General Medicine, Assam Medical College and Hospital, Dibrugarh, IND; 2 Department of Medicine, Assam Medical College and Hospital, Dibrugarh, IND; 3 Department of Radiology, Assam Medical College and Hospital, Dibrugarh, IND

**Keywords:** avascular necrosis (avn), avascular necrosis of the femoral head, hemophilia a, joint health, silent osteonecrosis

## Abstract

Avascular necrosis (AVN) of the femoral head is a rare but serious complication in patients with hemophilia. While hemarthroses commonly affect the knees, elbows, and ankles, hip involvement is less frequent and often overlooked, leading to delayed diagnosis, especially when presentation is silent. We report a case of a 32-year-old man with severe hemophilia A, diagnosed at age 11, who presented with progressive limp and right hip pain for six weeks. His treatment history revealed lifelong on-demand factor VIII replacement due to financial constraints, with multiple joint bleeds resulting in advanced hemophilic arthropathy. Despite a high Hemophilia Joint Health Score (HJHS) and a low Functional Independence Score in Hemophilia (FISH), he had never reported hip symptoms previously. Socioeconomic hardship, a lack of education, and complex family issues further hindered regular prophylaxis. Imaging with a computed tomography (CT) scan revealed avascular necrosis of the right femoral head. This case highlights the need for heightened awareness of silent AVN in persons with hemophilia, especially those on inadequate prophylaxis and with advanced joint disease. Routine hip screening should be a part of comprehensive musculoskeletal surveillance to detect early changes and enable timely intervention, ultimately preserving joint function and quality of life in resource-limited settings.

## Introduction

Hemophilia is an inherited bleeding disorder characterized by a deficiency of clotting factor VIII (hemophilia A) or IX (hemophilia B), leading to recurrent hemarthroses and progressive arthropathy in major joints such as the knees, elbows, and ankles. Hip joint involvement, although less frequent, can have profound clinical consequences due to the risk of avascular necrosis (AVN) of the femoral head, which remains an under-recognized but potentially debilitating complication in this population [1].

AVN, or osteonecrosis, results from compromised blood supply to the bone, leading to ischemia, bone infarction, and eventual structural collapse. While corticosteroid therapy, trauma, and alcohol use are well-known risk factors for femoral head AVN in the general population, the occurrence of AVN in patients with hemophilia is rare and often overlooked. Notably, some patients remain asymptomatic for prolonged periods, resulting in a “silent” presentation until significant structural damage has already occurred [2].

In hemophilia, repeated intra-articular bleeding may increase intra-capsular pressure, impairing the blood supply to the femoral head and predisposing to osteonecrosis [3]. However, because hip bleeds are less common than knee or ankle bleeds, AVN of the femoral head is often missed during routine evaluations focused on more typical target joints. Paton and Evans first described three cases of silent AVN of the femoral head in patients with hemophilia who did not present with typical hip pain, highlighting the importance of vigilant orthopedic assessment in this population [1].

More recent reports have reinforced this observation. For example, Kandzierski et al. (2021) described cases of femoral head necrosis in hemophilic patients, some of whom had no clear traumatic or corticosteroid-related etiology [4]. Similarly, Kemnitz et al. (2002) reported on the orthopedic challenges posed by AVN of the talus in children with hemophilia, emphasizing that osteonecrosis is not limited to the hip and may involve other weight-bearing joints [5]. A case report by Dandamudi et al. (2024) describes successful total hip arthroplasty (THA) in a patient with hemophilia B who developed femoral head AVN, underscoring the clinical significance and treatment complexity of this rare condition [6].

The early diagnosis of silent AVN in hemophilia remains challenging due to nonspecific or absent symptoms in the initial stages. Routine imaging, particularly magnetic resonance imaging (MRI), can detect early osteonecrotic changes before radiographic signs appear. Given the potential for hip joint destruction and disability, systematic surveillance of the hips, especially in patients with recurrent hemarthroses or prolonged factor deficiency, is advisable [5].

This case report aims to contribute to the limited body of evidence on silent AVN in patients with hemophilia by presenting a unique case of asymptomatic femoral head necrosis, which was identified during imaging performed as part of the evaluation for the patient’s symptoms. Such observations highlight the need for heightened clinical suspicion and multidisciplinary management to prevent irreversible joint damage and preserve quality of life.

## Case presentation

A 32-year-old man with known severe hemophilia A presented to the hemophilia outpatient department with complaints of persistent pain and progressive limp in his right hip for the past one and a half months. He described the pain as dull, deep-seated, and non-radiating, initially mild but progressively worsening to the point where he required support to walk short distances. The pain intensified with weight-bearing and prolonged standing and was partially relieved by rest and over-the-counter analgesics. He denied any history of significant trauma or fall preceding the onset of symptoms. Over the last two weeks, he noticed increasing difficulty in climbing stairs and getting up from a squatting position and severe discomfort while turning in bed at night. He also reported disturbed sleep due to nocturnal pain in the right hip.

The patient was diagnosed with severe hemophilia A (factor VIII deficiency of <1%) at the age of 11 years, following multiple episodes of spontaneous joint bleeds in his right knee and left elbow. Over the years, he has had frequent bleeding episodes involving the knees, elbows, and ankles, leading to progressive arthropathy and severe functional limitations. Due to financial constraints and the lack of access to uninterrupted factor replacement therapy, he has mostly relied on on-demand treatment, receiving factor VIII infusions only during major bleeds or significant swelling. He could not maintain a regular prophylactic regimen despite repeated counselling due to socioeconomic hardship. He denied any history of chronic corticosteroid use, alcohol abuse, hyperlipidemia, connective tissue disorders, or HIV infection. There was also no history suggestive of prior hip trauma or dislocation, although the latter could not be entirely excluded.

All routine laboratory investigations, including complete blood counts, liver and kidney function tests, lipid profile, thyroid function, and serology for HIV, were within normal limits (Table 1).

**Table 1:** Laboratory blood reports of the patient during hospital stay PCV, packed cell volume; MCV, mean corpuscular volume; MCH, mean corpuscular hemoglobin; MCHC, mean corpuscular hemoglobin concentration; SGOT, serum glutamic-oxaloacetic transaminase; ALT, alanine aminotransferase; SGOT, serum glutamic-pyruvic transaminase; AST, aspartate aminotransferase; HDL, high-density lipoprotein; VLDL, very low-density lipoprotein; LDL, low-density lipoprotein; TSH, thyroid-stimulating hormone; HbSAg, hepatitis B surface antigen; HCV, hepatitis C virus

Parameters	Values
Hemogram	
Hemoglobin	12.7 g/dL
Total Count	5,500/cmm
Differential Count	
Neutrophil	76%
Lymphocyte	17%
Monocyte	2%
Eosinophil	5%
Basophil	0%
Platelet Count	150,000
RBC Count	4.9 Million
PCV	41.6%
MCV	83.7 fL
MCH	25.6 pg
MCHC	35.5 g/dL
Liver Function Test	
Total Protein	7.42 g/dL
Albumin	4.30 g/dL
Total Bilirubin	0.77 mg/dL
SGOT (ALT)	32 U/L
SGPT (AST)	37 U/L
Alkaline Phosphatase	106 U/L
Lipid Profile	
Cholesterol	162 mg/dL
Triglycerides	133 mg/dL
HDL	35 mg/dL
VLDL	26.64 mg/dL
LDL	100 mg/dL
Kidney Function Test	
Urea	18.8 mg/dL
Creatinine	0.74 mg/dL
Sodium	137.82 mmol/L
Potassium	4.25 mmol/L
Thyroid Function Test	
TSH	2.38 µIU/mL
Infections	
Serology (HIV)	Non-reactive
HbSAg	Non-reactive
Anti-HCV	Non-reactive
Coagulation Profile	
Factor VIII	<1%
Inhibitor level	Not detected

On musculoskeletal examination during recent follow-ups, his Hemophilia Joint Health Score (HJHS) has consistently been high, reflecting advanced joint damage with limited range of motion and fixed deformities in both knees and elbows. His Functional Independence Score in Hemophilia (FISH) remains low, indicating significant restrictions in daily activities such as squatting, walking long distances, and performing household chores independently.

His educational and social background further complicated his disease management. He never attended formal schooling and has very limited health literacy. He lives with his elderly mother, who has a documented history of psychiatric illness requiring intermittent treatment. His father abandoned the family when the patient was around seven years old, leaving them dependent on irregular daily wage work and the support of distant relatives. These family circumstances have severely affected his ability to adhere to treatment protocols and to seek timely medical care for complications.

**Figure 1:**
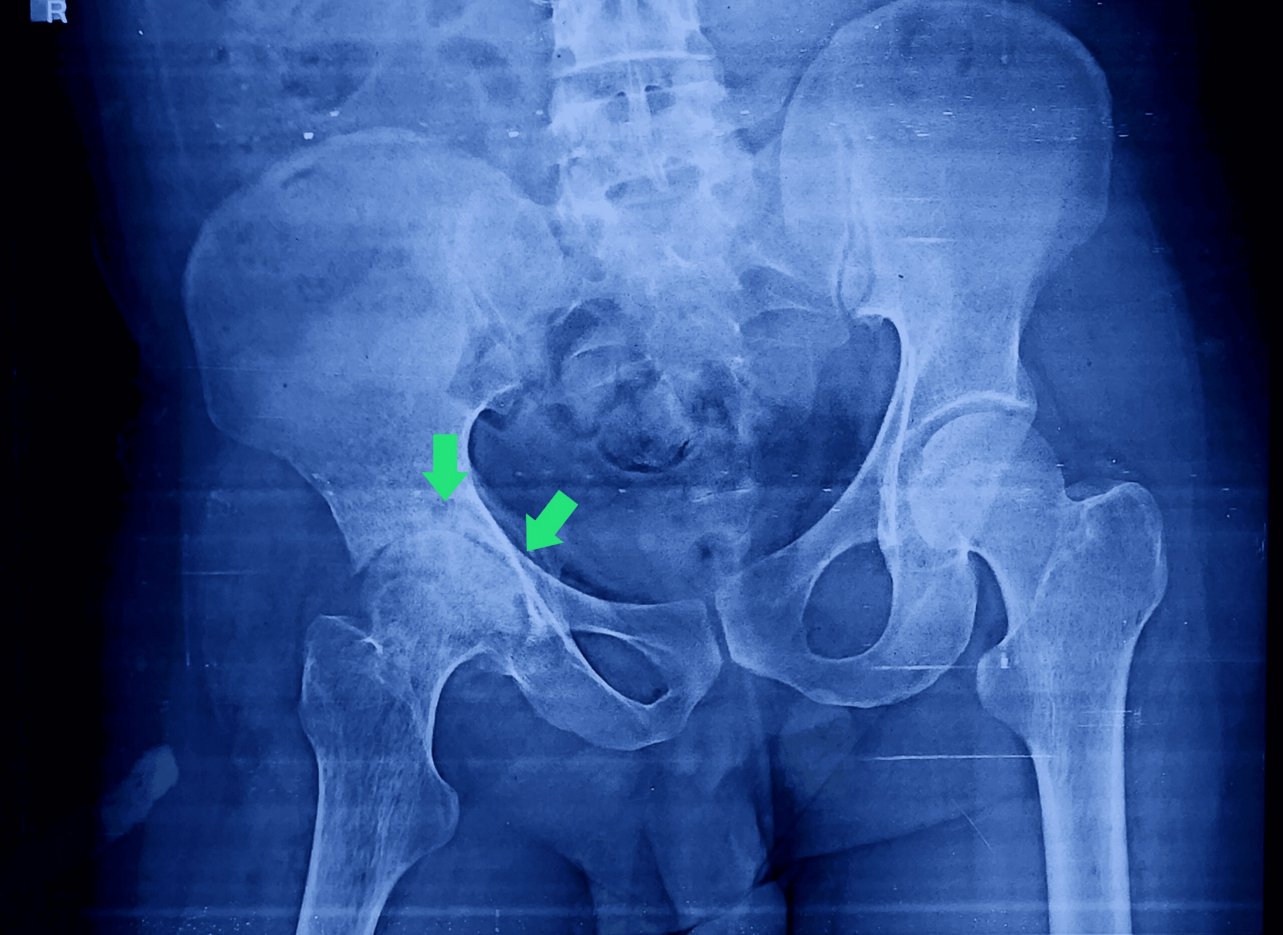
X-ray of the hip joint Mild flattening and sclerosis of the right femoral head, along with the reduction of joint spaces (green arrows)

In view of the patient’s persistent right hip pain and new-onset limp, an orthopedic consultation was sought for further evaluation. Initial plain radiographs of the pelvis and hips were obtained, which revealed subtle areas of increased radiodensity and irregularity in the contour of the right femoral head, raising suspicion for early avascular necrosis (Figure 1).

**Figure 2:**
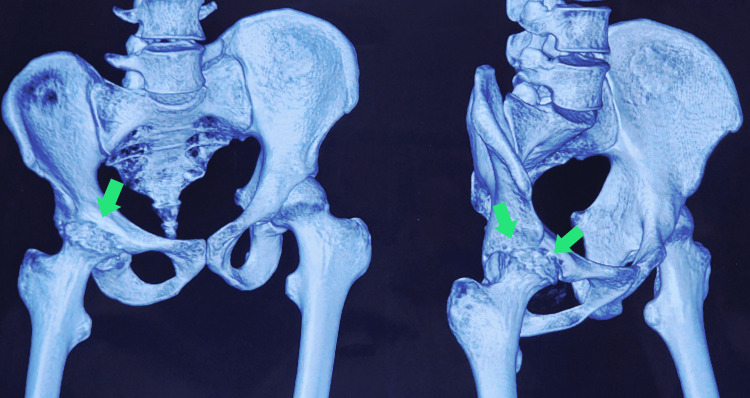
CT reconstruction of the hip joint The CT reconstruction image further confirms the findings of flattening and sclerosis of the right femoral head, suggestive of avascular necrosis (green arrows) CT: computed tomography

To assess the extent of bony involvement in greater detail, a computed tomography (CT) scan of the pelvis and hips was performed. The CT scan confirmed features consistent with the avascular necrosis of the right femoral head, demonstrating clear evidence of subchondral sclerosis, early crescent sign, and areas of focal collapse of the articular surface (Figure 2).

**Figure 3:**
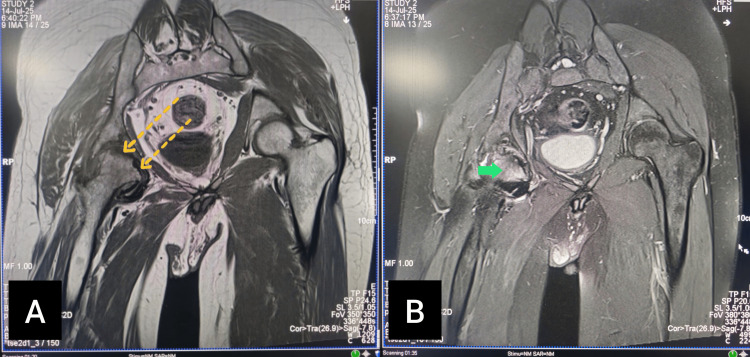
MRI of the right hip joint (A) Coronal T1 sequence reveals hypointensities in the subchondral region of the right femoral head (dotted yellow arrows). (B) Proton density fat-saturated (PDFS) sequence shows subchondral hyperintensities in the right femoral head (solid green arrow) MRI: magnetic resonance imaging

To further characterize the extent of marrow involvement and to detect any additional early changes that might not yet be visible on CT, a magnetic resonance imaging (MRI) of both hips was subsequently performed. The MRI revealed well-demarcated areas of low signal intensity on T1-weighted images and high signal intensity on T2-weighted images in the right femoral head, consistent with bone marrow edema and necrosis. The left femoral head appeared normal with no evidence of osteonecrosis or early marrow changes (Figure 3). The patient and his family were counselled about the diagnosis, the likely pathophysiological link to repeated intra-articular bleeds and raised intra-capsular pressure, and the natural course of the disease. A multidisciplinary management plan was advised, including orthopedic input for possible surgical options, structured physiotherapy for gait training and pain relief, and the optimization of factor VIII replacement therapy to prevent further joint damage.

## Discussion

Avascular necrosis (AVN), also known as osteonecrosis, is a pathological process characterized by the death of bone tissue due to a compromised blood supply, leading to the structural collapse of the affected bone and subsequent degenerative arthritis if left untreated. In the general population, AVN of the femoral head is most commonly associated with risk factors such as corticosteroid use, excessive alcohol intake, trauma, and certain systemic conditions such as sickle cell disease and systemic lupus erythematosus [7]. However, its occurrence in patients with hemophilia is rare and remains underreported in medical literature [1,2,4].

The pathophysiology of AVN in hemophilia is believed to differ from that in non-hemophilic individuals. Repeated intra-articular hemorrhages, a hallmark of hemophilic arthropathy, can lead to increased intra-capsular pressure within the hip joint, which may compress the retinacular vessels supplying the femoral head, ultimately resulting in ischemia and bone infarction [1,3]. Over time, the loss of viable bone and marrow tissue leads to subchondral collapse, joint incongruity, and secondary osteoarthritis. Importantly, this process may be insidious and clinically silent until significant damage has already occurred, as highlighted by Paton and Evans in their early description of “silent” AVN in patients with hemophilia [1].

Although hemophilic arthropathy most commonly affects the knees, elbows, and ankles due to repeated bleeds into these target joints, the hip joint can also be involved but is often overlooked during routine musculoskeletal assessments [2,4]. Published case series suggest that the prevalence of femoral head AVN in hemophilia is very low, estimated at around 2%-3% in some cohorts [1,4]. More recent case reports, such as that by Dandamudi et al. (2024), indicate that AVN can occur in both hemophilia A and B and may present significant challenges for surgical management due to the inherent bleeding risk [6].

The prevention of AVN in hemophilia primarily hinges on optimal bleed control. Prophylactic factor replacement therapy has been shown to dramatically reduce the frequency of spontaneous bleeds and prevent progressive joint damage [8]. However, financial and logistical barriers often limit access to continuous prophylaxis in resource-constrained settings, as was evident in this patient’s history. Comprehensive care, including the early diagnosis and regular monitoring of all major joints, is critical. Hip joint examination should not be neglected, particularly in patients with high Hemophilia Joint Health Scores (HJHS) and signs of gait disturbance or unexplained hip pain [1,6].

Once AVN is established, management requires a multidisciplinary approach. Early stages may be amenable to conservative measures such as protected weight-bearing, physiotherapy, analgesia, and core decompression procedures [9]. However, advanced stages with structural collapse often necessitate surgical intervention, most commonly total hip arthroplasty (THA). In patients with hemophilia, THA is feasible and has been shown to improve pain and mobility outcomes when performed with meticulous perioperative factor replacement and hematology-orthopedic collaboration [6,10].

Given the significant risk of surgical bleeding and post-operative complications, perioperative factor coverage must be tailored individually to maintain adequate factor levels before, during, and after surgery. Post-operative rehabilitation is equally important to restore joint function and prevent further musculoskeletal disability [10].

## Conclusions

In summary, AVN of the femoral head, though rare in hemophilia, poses significant challenges due to its silent nature and the complex interplay between underlying arthropathy, socioeconomic barriers, and limited access to prophylaxis. Vigilant joint monitoring, patient education, accessible prophylactic regimens, and a robust multidisciplinary care framework remain the cornerstones for the early detection, prevention, and effective management of this disabling complication in persons with hemophilia.

## References

[1] Paton RW, Evans DI (1988). Silent avascular necrosis of the femoral head in haemophilia. J Bone Joint Surg Br.

[2] Kilcoyne RF, Nuss R (1999). Femoral head osteonecrosis in a child with hemophilia. Arthritis Rheum.

[3] MacNicol MF, Ludlam CA (1999). Does avascular necrosis cause collapse of the dome of the talus in severe haemophilia?. Haemophilia.

[4] Kandzierski G, Matuszewski Ł, Stec S (2021). Aseptic necrosis of the femoral head in the course of haemophilia; a different clinical and radiological course compared to typical Legg-Calve-Perthes disease (twenty years of follow-up) - case report. Chir Narzadow Ruchu Ortop Pol.

[5] Kemnitz S, Moens P, Peerlinck K, Fabry G (2002). Avascular necrosis of the talus in children with haemophilia. J Pediatr Orthop B.

[6] Dandamudi S, Tseng J, Ratz J, Boggio L, Levine BR (2024). Total hip arthroplasty for avascular necrosis in a patient with hemophilia B. Arthroplast Today.

[7] Mont MA, Jones LC, Hungerford DS (2006). Nontraumatic osteonecrosis of the femoral head: ten years later. J Bone Joint Surg Am.

[8] Srivastava A, Santagostino E, Dougall A (2020). WFH guidelines for the management of hemophilia, 3rd edition. Haemophilia.

[9] Lieberman JR, Berry DJ, Mont MA, Aaron RK, Callaghan JJ, Rajadhyaksha AD, Urbaniak JR (2003). Osteonecrosis of the hip: management in the 21st century. Instr Course Lect.

[10] Mortazavi SJ, Daneshpoor SM, Shafiei SH (2023). Total hip arthroplasty in patients with hemophilia: what do we know?. Arch Bone Jt Surg.

